# Negative Prognostic Implication of *TERT* Promoter Mutations in Human Papillomavirus–Negative Tonsillar Squamous Cell Carcinoma Under the New 8th AJCC Staging System

**DOI:** 10.1007/s13193-020-01200-9

**Published:** 2020-09-05

**Authors:** Hyunchul Kim, Mi Jung Kwon, Bumjung Park, Hyo Geun Choi, Eun Sook Nam, Seong Jin Cho, Kyueng-Whan Min, Eun Soo Kim, Hee Sung Hwang, Mineui Hong, Taeryool Koo, Hyo Jung Kim

**Affiliations:** 1 Department of Pathology, Dongtan Sacred Heart Hospital, 7, Keunjaebong-gil, Hwaseong-si, Gyeonggi-do 18450 Republic of Korea; 2 Department of Pathology, Hallym University Sacred Heart Hospital, Hallym University, 22, Gwanpyeong-ro 170beon-gil, Dongan-gu, Anyang-si, Gyeonggi-do 14068 Republic of Korea; 3 Department of Otorhinolaryngology-Head and Neck Surgery, Hallym University Sacred Heart Hospital, Hallym University College of Medicine, Anyang, Republic of Korea; 4 Department of Pathology, Kangdong Sacred Heart Hospital, Hallym University College of Medicine, Seoul, 134-701 Republic of Korea; 5 Department of Pathology, Hanyang University Guri Hospital, Hanyang University College of Medicine, Kyoungchun-ro 153, Guri-si, Gyeonggi-do 11923 Republic of Korea; 6 Department of Radiology, Hallym University Sacred Heart Hospital, Hallym University College of Medicine, Anyang, Republic of Korea; 7 Department of Nuclear Medicine, Hallym University Sacred Heart Hospital, Hallym University College of Medicine, Anyang, Republic of Korea; 8 Department of Pathology, Kangnam Sacred Heart Hospital, Hallym University College of Medicine, Daerim 1–Dong, Yeongdeungpo-gu, Seoul, 150-950 Republic of Korea; 9 Department of Radiation Oncology, Hallym University Sacred Heart Hospital, Hallym University College of Medicine, Anyang, Republic of Korea; 10 Department of Hematological Oncology, Hallym University Sacred Heart Hospital, Hallym University College of Medicine, Anyang, Republic of Korea

**Keywords:** Tonsil, Squamous cell carcinoma, Human papillomavirus, Head and neck cancer, Prognosis

## Abstract

Telomerase reverse transcriptase gene promoter (*TERTp*) mutation is a potential candidate for pathogenesis and therapeutic target of tonsillar squamous cell carcinomas (TSCCs) in association with human papillomavirus (HPV). Their clinical relevance has not been validated under the new 8th American Joint Committee on Cancer (AJCC) staging system. We analyzed real-time peptide nucleic acid–mediated PCR and sequencing methods (*TERTp* mutation) and real-time PCR-based assay (HPV) in 80 surgically resected TSCCs. The 8th edition staging system improved the stratification of the early and advanced stages and between T or N categories for overall survival over the 7th edition. *TERTp* mutation was found in 7.5%, and HPV in 80.0% of the patients. The majority (83.3%) of *TERTp* mutation cases were HPV-positive TSCCs. Applying the 8th edition staging system, *TERTp* mutation was an independent factor of poor prognosis for disease-free survival (DFS) in TSCC patients, supporting the clinical significance of *TERTp* mutation in tonsil cancer. *TERTp* mutations were also negatively correlated with overall survival and DFS in HPV-negative TSCCs. Conclusively, *TERTp* mutation provides negative prognostic impact on survival of surgically managed tonsil cancers staged with the AJCC 8th edition.

## Introduction

Tonsillar squamous cell carcinomas (TSCCs) account for 70–80% of the oropharyngeal cancers most prevalent for human papillomavirus (HPV) [1–3]. In oropharyngeal squamous cell carcinomas (SCCs), HPV is associated with better prognosis and response to radiochemotherapy compared with HPV-negative oropharyngeal SCCs [3, 4]. Tonsils are the subsite of oropharyngeal cancers with the highest HPV-positive rate [2, 3]. The 5-year survival in early cases has been reported to be > 90%, which decreases to < 20% in advanced tonsil cancer [1–3, 5]. Both primary surgery and radiotherapy/chemotherapy are effective treatments for TSCCs [4]; however, treatment failures can develop unexpectedly. The 8th edition of the American Joint Committee on Cancer (AJCC) staging system for oropharyngeal cancer incorporates HPV infection and extranodal extension [6], but it is unclear whether those result in better stratification of Korean patients, as the area has relatively low incidence of oropharyngeal cancer [7–9]. Because risk stratification, prognosis prediction, treatment selection, and follow-up strategies often depend on the AJCC system, validation of staging is crucial.

The *TERT* promoter (*TERTp*), a critically important regulatory element for telomerase expression harboring binding sites for a number of transcriptional activators and repressors, contributes to increased telomerase activity that leads to immortalization of cells, which is one of the hallmarks of cancer [10, 11]. Two mutually exclusive G–A mutations at nucleotide − 124 and − 146 within the core promoter region of *TERT* gene occur as a pathogenic mutation in human malignancies [12], which are also driver mutations in head and neck SCCs [13–17]. The *TERTp* mutation, alone and with the HPV oncogenes, plays an important role in oral and uterine cervical SCCs [14, 18, 19]. Two viral oncogenes, E6 and E7, expressed by high-risk HPV-associated cancers, affect the oncogenic pathway related to cellular immortalization, typically activating telomerase expression [18, 20, 21]. Although previous studies examined multiple subsites of oral or oropharyngeal cancers, the frequencies of *TERTp* mutation in tonsil cancers and the association to HPV have rarely been investigated in the series of their studies [22, 23]. Furthermore, the revised 8th AJCC staging system in terms of oropharyngeal cancers with or without HPV has not been validated in the Far East Asian cohort data, specifically on tonsil cancers.

Here, we investigated *TERTp* mutation, HPV infection, and clinicopathological characteristics in 80 Korean primary TSCC patients. We sought to validate the 8th edition of the AJCC staging system compared with the 7th edition, with variation in patient location and therapy.

## Patients and Methods

### Patients

Formalin-fixed, paraffin-embedded (FFPE) tissues were obtained from 80 TSCC patients who underwent primary resection, with no prior treatment and complete medical records at our institution between 1997 and 2018. Clinical information was analyzed using medical records and radiological results. Heavy smoking was defined as > 20 pack-years [6]. Alcohol consumption was defined as > 14 drinks/week [6]. Of these 80 patients, 11 patients underwent postoperative radiotherapy, 2 patients had chemotherapy, and 39 patients had chemoradiotherapy following the surgical resection. The remaining 28 patients were treated with surgery alone. Radiation doses ranged from 5040 to 7200 cGy/36 fractions over 8 weeks.

Diagnosis and histological differentiation were evaluated according to the World Health Organization classification [1]. Patients were re-staged according to the 8th editions of the AJCC/UICC TNM classification [6]. The study protocol was approved by Sacred Heart Hospital Institutional Review Board (No. 14-2-57) and performed in accordance with the relevant guidelines and regulations (Declaration of Helsinki). Informed consent was obtained from the patients and from the next of kin (deceased patients) before enrollment in the study.

### DNA Extraction and Detection of TERT Promoter Mutation

Genomic DNA was extracted from 10-μm-thick sections of 10% neutral FFPE tumor tissue blocks using Maxwell 16 FFPE Tissue LEV DNA Purification Kit for DNA (Promega, USA). *TERTp* mutations (C250 and C228) were identified using the PNAClamp™ TERT mutation detection kit (PANAGENE, Daejeon, South Korea [10]. Subsequently, the *TERTp* mutation analyses were also confirmed by directional sequencing of PCR fragments amplified from genomic DNA. The primers used for *TERTp* were as follows [24]: forward, 5′-AGTGGATTCGCGGGCACAGA-3′, and reverse, 5′-AGCACCTCGCGGTAGTGG-3′, which amplified a 346 bp fragment. PCR amplification was carried out in a reaction volume of 30 μl containing 100 ng of template DNA, 10× PCR buffer, 0.25 mM dNTPs, 10 pmol primers, and 1.25 U Taq DNA polymerase (Solgent, Korea). The thermal cycling conditions were as follows: denaturation at 95 °C for 3 min, followed by 10 cycles of 95 °C denaturation for 30 s, 60 °C annealing for 30 s, and 68 °C elongation for 1 min. This was followed by 30 cycles under the same settings, with the elongation step modified to continue for an additional 5 s each cycle. PCR was completed with final elongation at 68 °C for 7 min. PCR products were electrophoresed on 2% agarose gels and purified with a Solgent PCR purification kit (Solgent). All amplification products were sequenced bidirectionally using an automated sequencer (ABI 3130xl; Applied Biosystems, Foster City, CA, USA) using the BigDye Terminator v1.1 kit (Applied Biosystems) and the appropriate forward and reverse primers.

### Detection of HPV

HPV status was evaluated by PANA RealTyper HPV genotyping kit and PANA RealTyper HPV screening kit (PANAGENE). This kit, approved for clinical use in Korea, detects a total of 40 HPV genotypes including 20 high-risk genotypes (16, 18, 26, 31, 33, 35, 39, 45, 51, 52, 53, 56, 58, 59, 66, 68, 69, 70, 73, and 82), 2 low-risk genotypes (6 and 11), and 18 other genotypes. Briefly, real-time PCR assays were performed in a 25 μl reaction mixture containing 19 μl of HPV mix, 1 μl of Taq DNA polymerase, and 5 μl of extracted DNA, positive control, or negative control. PCR was performed using the following conditions: 1 cycle of incubation at 50 °C for 2 min and Taq activation at 95 °C for 15 min; 45 cycles of denaturation at 95 °C for 15 s, annealing at 55 °C for 45 s, and extension at 72 °C for 15 s; and a melting curve step at 95 °C for 5 min, 35 °C for 5 min, followed by increase in temperature from 35 °C to 80 °C for 5 min, with a gradual increment of 0.5 °C (every 5 s) to achieve fluorescence in all four channels (FAM, HEX or VIC, ROX, and Cy5).

### Statistical Analysis

Correlations between the *TERTp* mutation and clinicopathological variables were assessed using the Chi-squared test or two-tailed Fisher’s exact test. Factors found to be significant in univariate analysis were included in subsequent binary logistic regression analysis to identify independent variables associated with *TERTp* mutation. Survival analyses were performed using the Kaplan-Meier method and were compared using a log-rank test. Overall survival (OS) was defined as the interval from the first day of surgery until death. Disease-free survival (DFS) was defined as the interval from the first day of surgery until tumor recurrence. OS and DFS were analyzed until February 2019. Univariate and multivariate analyses using the Cox proportional hazard regression model were applied to determine the hazard ratio (HR) and 95% confidence intervals (CI) for specific variables related to OS and DFS. SPSS version 18 (SPSS Inc., Chicago, IL, USA) was used for all statistical analyses. *P* values < 0.05 were considered statistically significant.

## Results

### Comparisons Between the AJCC 8th and 7th Edition Staging Systems

According to the 8th edition staging system, 18 (22.5%) tumors were classified as T1, 31 (38.8%) as T2, 20 (25.0%) as T3, and 11 (13.7%) as T4. Of the 80 patients, 17 (21.3%) were categorized as N0, 37 patients (46.3%) as N1, 11 (13.7%) as N2, and 15 (18.7%) as N3. Combining the T and N categories, the overall stages of 31 patients were diagnosed as stage I (38.8%), 18 (22.5%) as stage II, 11 (13.7%) as stage III, and 20 (25.0%) as stage IV. The median follow-up period was 64 months (range, 3–136 months). The 5-year OS and DFS rates were 53.2% and 43.8%, respectively.

**Fig. 1:**
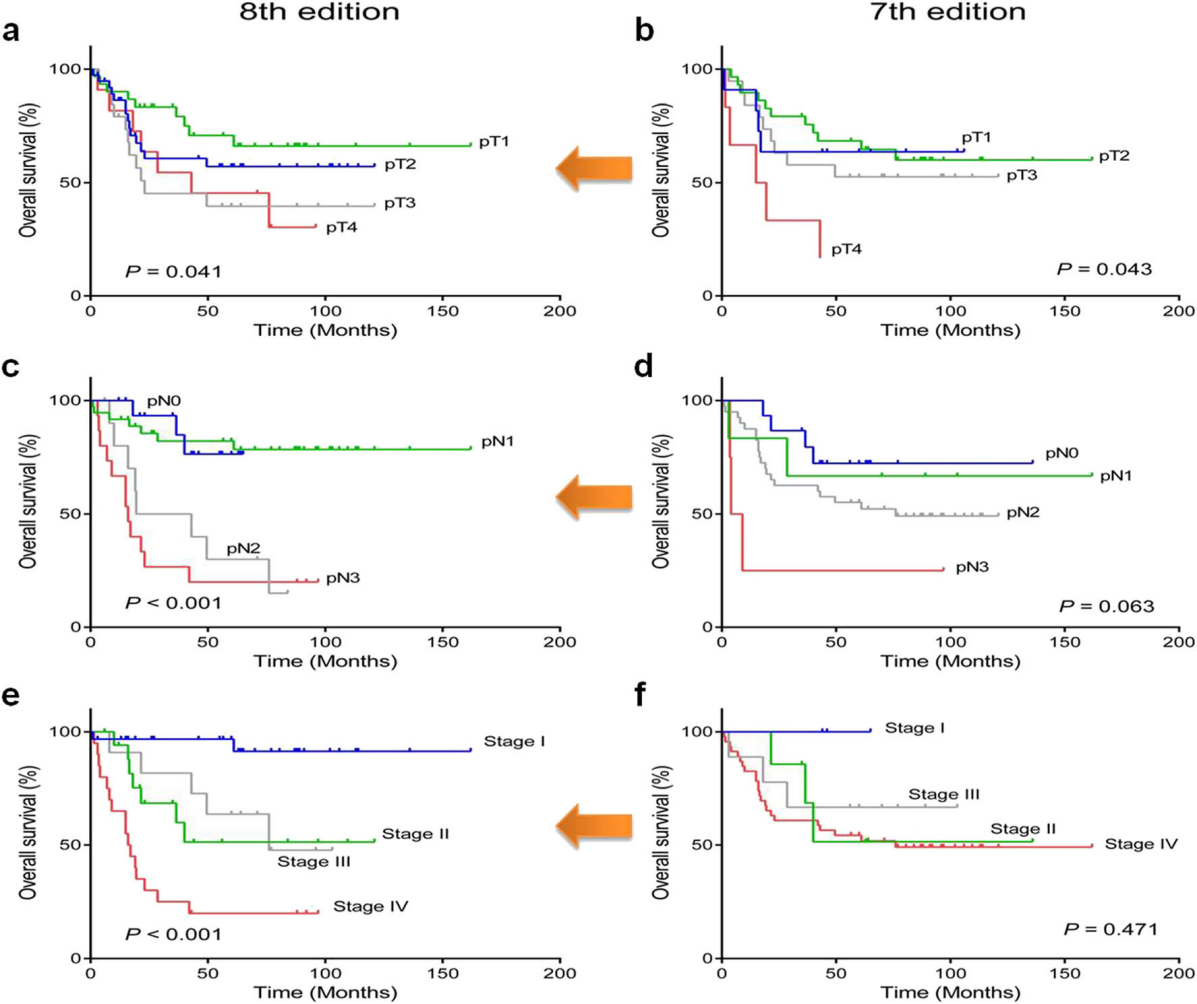
Overall survival analyses of tonsillar squamous cell carcinomas according to newly revised pT category (**a**), pN category (**c**), and AJCC stage 8th (**e**) compared with previous pT (7th edition) (**b**), pN (7th edition) (**d**), and AJCC stage 7th (**f**)

We performed Kaplan–Meier survival analyses of OS in 80 patients who had previous TNM information according to AJCC 8th vs. AJCC 7th staging system (Fig. 1). We compared the T category, N category, and overall stages assigned by the 8th and 7th editions of the AJCC staging system. As shown in Fig. 3, the 7th edition of the AJCC staging system performed poorly with respect to the discrimination and stratification of N category and overall stages (*P* = 0.063 and *P* = 0.471, respectively). Only the T category was well discriminated according to clinical outcomes (*P* = 0.043). In contrast, the 8th edition provided statistically significant stratification for the T category, N category, and overall staging (*P* = 0.041, *P* < 0.001, and *P* < 0.001, respectively). Therefore, the 8th edition was applied in our study.

### HPV and *TERTp* Mutation

**Fig. 2:**
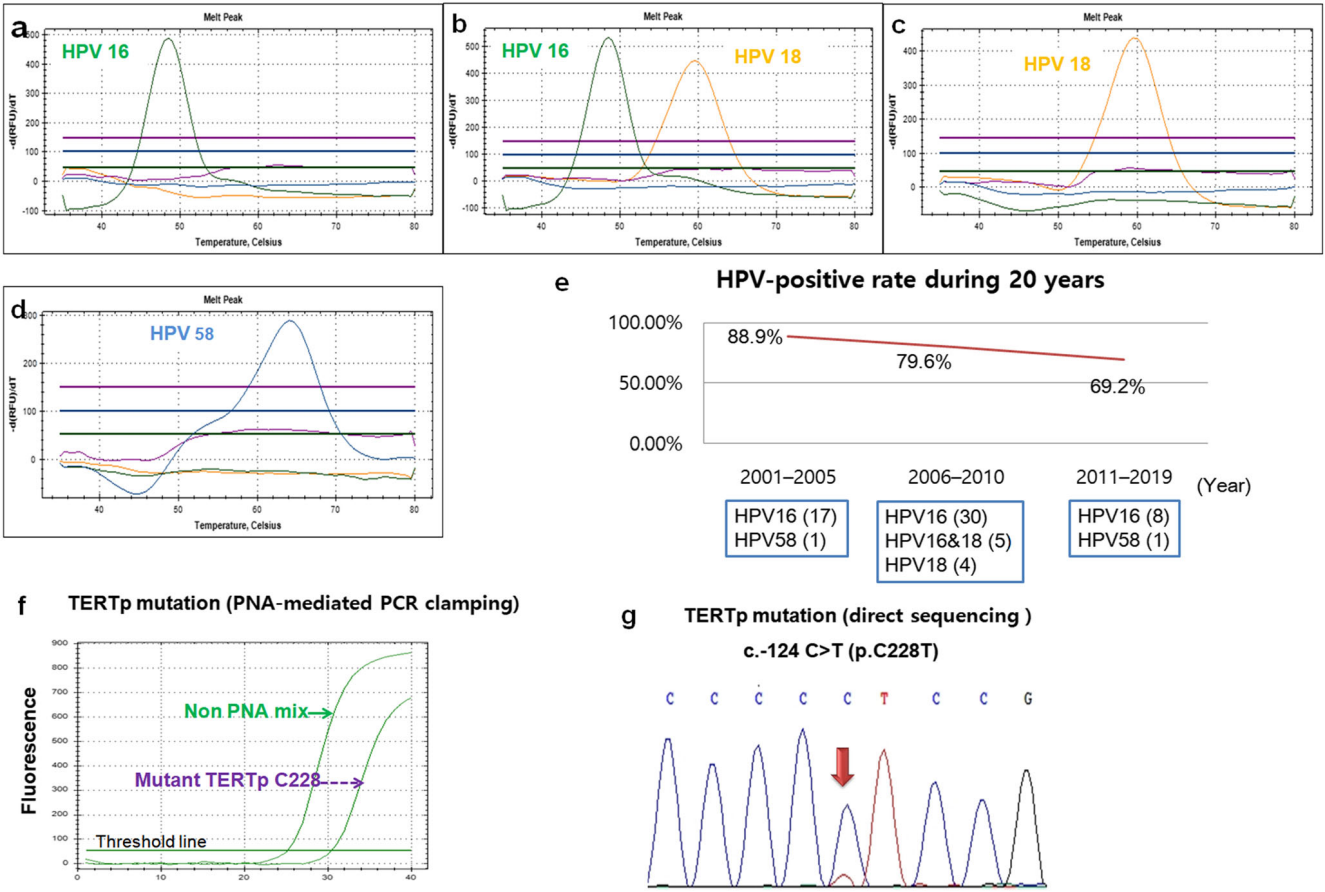
(**a**–**c**) HPV genotypes detected in tonsillar squamous cell carcinomas: HPV 16 (**a**), concurrent HPV 16 and HPV 18 (**b**), and HPV 18 (**c**). **d** HPV-negative tonsil cancers. **e** The periodic incidence of HPV in tonsillar squamous cell carcinomas during 20 years. **f** PNA clamp real-time PCR detected *TERTp* mutation on C228 in tonsillar squamous cell carcinomas. **g** Sequence chromatography demonstrated *TERTp* c.-124 C > T (p. C228T) mutation

HPV was identified in 64 tumors (80.0%) analyzed by real-time HPV genotyping kit. All 80 cases were interpretable. There were only high-risk HPV genotypes including HPV 16 (54/64, 84.4%), HPV 18 (4/64, 6.2%), HPV 58 (1/64, 1.6%), and concurrent HPV 16 and HPV 18 (5/64, 7.8%) (Fig. 2a–d). We also analyzed the incidence of HPV in TSCCs during the last 20 years (Fig. 2e). The HPV-positive rates in those tonsil cancers decreased from 88.9% (16/18) in 2001–2005 to 79.6% (29/49) in 2006–2010 and 69.2% (9/13) in 2011–2019.

The real-time quantitative PCR with PNA-mediated clamping method identified six mutations at position − 124 within *TERTp* in 80 TSCC patients (Fig. 2f). No *TERTp* mutation at position − 146 was identified. To confirm these results, direct sequencing was repeatedly performed and consistently identified *TERTp* mutations in 6 (7.5%) of 80 tumor samples. The observed point mutations were hot spot nucleotide changes G to A at position − 124 within *TERTp* in 6 cases (Fig. 2g).

We analyzed the associations of HPV or *TERTp* mutation with clinical and pathological features of 80 TSCCs (Table 1). The presence of HPV was more frequently associated with younger age (≤ 60 years) (*P* = 0.010), low alcohol consumption (*P* = 0.021), pN-positive status (*P* < 0.001), lower AJCC stage (*P* = 0.020), and presence of ipsilateral lymph node metastasis (*P* = 0.004). HPV positivity was not associated with *TERTp* mutation. There were no statistical associations between *TERTp* mutation and clinicopathologic features of TSCCs.

**Table 1:** Association between HPV and *TERTp* mutation and patient characteristics

Parameter	Total	HPV			*TERTp*		
		Positive	Negative	*P*	Mutated	Wildtype	*P*
	*N* = 80 (%)	*n* = 64 (80.0%)	*n* = 16 (20.0%)		*n* = 6 (7.5%)	*n* = 74 (92.5%)	
Sex				1.000			1.000
Male	70 (87.5)	56 (87.5)	14 (87.5)		6 (100)	64 (86.5)	
Female	10 (12.5)	8 (12.5)	2 (12.5)		0 (0)	10 (13.5)	
Age (year)				0.010*			1.000
≤ 60	52 (65.0)	46 (71.9)	6 (37.5)		4 (66.7)	48 (64.9)	
> 60	28 (35.0)	18 (28.1)	10 (62.5)		2 (33.3)	26 (35.1)	
Smoking				0.263			0.392
Light	33 (41.3)	32 (50.0)	5 (31.2)		1 (16.7)	32 (43.2)	
Heavy	47 (58.7)	32 (50.0)	11 (68.8)		5 (83.3)	42 (56.8)	
Alcohol				0.021*			0.667
Light	50 (62.5)	44 (68.8)	6 (37.5)		3 (50.0)	47 (63.5)	
Heavy	30 (37.5)	20 (31.2)	10 (62.5)		3 (50.0)	27 (36.5)	
Tumor location				0.173			0.651
Right side	47 (58.7)	40 (62.5)	7 (43.8)		3 (50.0)	44 (59.5)	
Left side	33 (41.3)	24 (37.5)	9 (56.2)		3 (50.0)	30 (40.5)	
pT category				0.088			0.556
T1-T2	49 (61.3)	36 (56.3)	13 (81.3)		3 (50.0)	46 (62.2)	
T3-T4	31 (38.7)	28 (43.7)	3 (18.7)		3 (50.0)	28 (37.8)	
pNodal status				< 0.001*			0.333
N0	17 (21.3)	8 (12.5)	9 (56.2)		0 (0)	17 (23.0)	
N1–3	63 (78.7)	56 (87.5)	7 (43.8)		6 (100)	57 (77.0)	
pAJCC stage (8th)				0.020*			0.624
I–III	60 (75.0)	52 (81.2)	8 (50.0)		4 (66.7)	56 (75.7)	
IV	20 (25.0)	12 (18.8)	8 (50.0)		2 (33.3)	18 (24.3)	
HPV status				–			1.000
Positive	64 (80.0)	–	–		5 (83.3)	59 (79.7)	
Negative	16 (20.0)	–	–		1 (16.7)	15 (20.3)	
BOT invasion				0.263			0.407
Present	43 (53.8)	32 (50.0)	5 (31.2)		4 (66.7)	33 (44.6)	
Absent	37 (46.2)	32 (50.0)	11 (68.8)		2 (33.3)	41 (55.4)	
Soft palate invasion				0.154			0.423
Present	28 (35.0)	25 (39.1)	3 (18.7)		3 (50.0)	25 (33.8)	
Absent	52 (65.0)	39 (60.9)	13 (81.3)		3 (50.0)	49 (66.2)	
Ipsilateral LN meta				0.004*			1.000
Present	58 (72.5)	51 (79.7)	7 (43.8)		5 (83.3)	53 (71.6)	
Absent	22 (27.5)	13 (20.3)	9 (56.2)		1 (16.7)	21 (28.4)	
Contralateral LN meta				1.000			1.000
Present	12 (15.0)	10 (15.6)	2 (12.5)		1 (16.7)	11 (14.9)	
Absent	68 (85.0)	54 (84.4)	14 (87.5)		5 (83.3)	63 (85.1)	
ENE				0.485			0.409
Present	51 (63.8)	42 (65.6)	9 (56.2)		5 (83.3)	46 (62.2)	
Absent	29 (36.2)	22 (34.4)	7 (43.8)		1 (16.7)	28 (37.8)	

*HPV* human papillomavirus, *p* pathologic, *LN* lymph node, *BOT* base of tongue, *AJCC* American Joint Committee on Cancer, *ENE* extranodal extension

*Statistically significant, *P* < 0.05

**Fig. 3:**
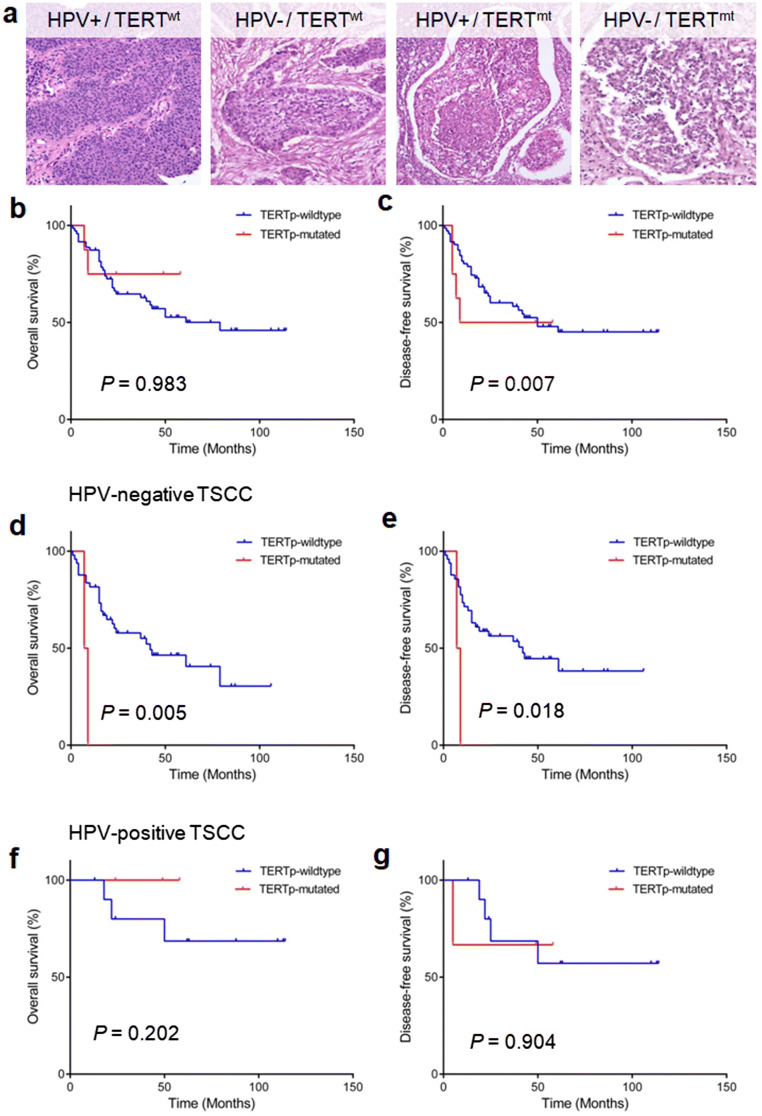
**a** Representative H&E images according to HPV and *TERTp* mutation (magnifications, × 200). *TERTp* mutation is not related to overall survival (**b**), whereas it has a strong prognostic impact on shorter disease-free survival (**c**). In HPV-negative tumors, *TERTp* mutation is associated with worse overall survival (**d**) and disease-free survival (**e**). However, there are no survival correlations in terms of overall (**f**) and disease-free (**g**) survivals in HPV-positive tumors

Coexistence of HPV and *TERTp* mutation occurred in 5 cases (6.3%) of 80 TSCCs: a total of 5 cases (83.3%) among the 6 *TERTp* mutations were in HPV 16-positive tonsil cancers and one *TERTp* mutated tumor was HPV-negative. TSCCs showed the most frequently HPV-positive/*TERTp*-wild-type (*n* = 59, 73.7%), followed by HPV-negative/*TERTp*-wild-type (*n* = 15, 18.8%), HPV-positive/*TERTp*-mutated (*n* = 5, 6.2%), and HPV-negative/*TERTp*-mutated (*n* = 1, 1.3%) (Fig. 3a).

### Prognostic Correlation of *TERTp* Mutation

In Kaplan–Meier survival analyses, *TERTp* mutation was found to be statistically associated with shorter DFS rates than those of *TERTp* wild-type (*P* = 0.007), whereas no statistical difference was observed for OS between *TERTp* mutation and *TERTp* wild-type (*P* = 0.983) (Fig. 3b, c). We further analyzed the prognostic impact of *TERTp* mutations on OS and DFS according to HPV status. *TERTp* mutations were strongly correlated with decreased OS and DFS in patients with HPV-negative TSCCs (*P* = 0.005 and *P* = 0.018, respectively) (Fig. 3d, e). However, there were no prognostic correlations of *TERTp* mutations with OS or DFS *TERTp* mutations in patients with HPV-positive TSCCs (*P* = 0.202 and *P* = 0.904, respectively) (Fig. 3f, g).

We analyzed the OS and DFS through univariate and multivariate analyses (Table 2). In the univariate analyses, older age (*P* = 0.006), higher T category (*P* = 0.007), and base of tongue (BOT) invasion (*P* = 0.015) were associated with shorter OS rates, while *TERTp* mutation (*P* = 0.007), higher T category (*P* = 0.004), BOT invasion (*P* = 0.002), and soft palate invasion (*P* = 0.005) were associated with shorter DFS rates. Multivariate analyses confirmed that older age and higher T category were independent negative prognostic factors for shorter OS in patients with TSCCs (*P* < 0.001, HR: 4.467, 95% CI: 2.037–9.793; *P* = 0.016, HR: 3.152, 95% CI: 1.244–7.988, respectively). *TERTp* mutation was identified to be the only independent prognostic factor for DFS in tonsil cancers (*P* = 0.021, HR: 3.216, 95% CI: 1.197–8.644).

**Table 2:** Univariate and multivariate analyses of overall survival and disease-free survival of patients with tonsillar squamous cell carcinoma by univariate and multivariate analyses

	Overall survival				Disease-free survival			
	Univariate		Multivariate		Univariate		Multivariate	
	HR (95% CI)	*P*	HR (95% CI)	*P*	HR (95% CI)	*P*	HR (95% CI)	*P*
*TERTp*	0.984	0.983			3.879	0.007*	3.216	0.021*
Wildtype vs. mutated	(0.234–4.146)				(1.449–10.388)		(1.197–8.644)	
HPV	0.580	0.195			0.802	0.567		
Absent vs. present	(0.255–1.321)				(0.377–1.707)			
Sex	0.454	0.282			0.719	0.533		
Male vs. female	(0.108–1.910)				(0.255–2.029)			
Age (year)	2.744	0.006*	4.467	< 0.001*	1.813	0.075		
< 60 vs. ≥ 60	(1.327–5.674)		(2.037–9.793)		(0.943–3.488)			
Tonsil side	1.396	0.363			0.917	0.794		
Rt vs. Lt	(0.680–2.863)				(0.478–1.758)			
Alcohol	1.371	0.394			1.064	0.852		
Light vs. heavy	(0.663–2.834)				(0.554–2.045)			
Smoking	1.853	0.105			1.358	0.353		
Light vs. heavy	(0.878–3.908)				(0.712–2.591)			
pT category	2.745	0.007*	3.152	0.016*	2.549	0.004*	1.556	0.257
T1–2 vs. T3–4	(1.321–5.708)		(1.244–7.988)		(1.340–4.850)		(0.725–3.341)	
pN category	2.651	0.110			2.821	0.050		
N0 vs. N1–3	(0.801–8.773)				(0.998–7.968)			
BOT invasion	2.579	0.015*	1.770	0.222	2.951	0.002*	1.823	0.159
Absent vs. present	(1.206–5.513)		(0.707–4.426)		(1.504–5.791)		(0.790–4.206)	
Soft palate invasion	1.775	0.116			2.513	0.005*	1.445	0.356
Absent vs. present	(0.867–3.630)				(1.323–4.771)		(0.662–3.156)	

*HR* hazard ratio, *CI* confidence interval, *HPV* human papillomavirus, *Rt* right, *Lt* left, *p* pathologic, *BOT* base of tongue

*Statistically significant, *P* value < 0.05

## Discussion

In the present study, the new staging system improved stratification of early and advanced stages and between T or N category concerning overall survival based on Korean population. Under the new AJCC 8th edition, the prognostic implication of *TERTp* mutation in tonsil cancers has been rarely reported. Applying the new AJCC 8th edition, we confirmed the negative prognostic impact of *TERTp* mutation on survival of surgically managed tonsil cancers staged with the new AJCC 8th edition, especially on HPV-negative TSCCs. *TERTp* mutation has been reported as a predictor of poor prognosis in laryngeal cancers but not in oral SCCs [17, 25]. A meta-analysis based on published articles concluded that *TERTp* mutation serves as an adverse prognostic factor in any cancer regardless of organ [26]. Overexpression of *TERT* by its promoter mutation representing late events of the oncogenic process may increase the self-renewal capacity of cancer stem cells and induce poor clinical outcomes [27, 28]. The non-canonical functions of *TERTp* mutation might biologically sustain how *TERTp* mutation is related to poor prognosis.

Vinagre et al. [29] divided various tumors into those with a high frequency of mutations (≥ 5%) and tumors with no mutations or with a very low frequency of *TERTp* mutations (< 5%) because mutations affecting the telomerase coding region are very uncommon in the cancer setting [11]. We observed 7.5% *TERTp* mutation in TSCCs, which is a relatively high frequency according to Vinagre et al. [29]. The frequency of *TERTp* mutation is variable in head and neck SCCs, which could be because the *TERTp* mutation can also result from environmental factors such as ultraviolet radiation and chemical carcinogens [29, 30]. Thus, oral cavity SCCs have a wide range of the frequency of 0–65% for the *TERTp* mutation [14, 17, 22, 23]; this mutation is also common for tongue area (63.6%) and laryngeal SCCs (27%) [22, 25].

The lack of specific clinicodemographic features related to *TERTp* mutation, found in the present study, has been mainly described in SCCs in head and neck or other sites including skin, lung, and uterine cervix [14, 22, 25, 31]. The majority (83.3%) of *TERTp* mutations occurred exclusively in the HPV16-positive TSCCs, although there was no statistical association between *TERTp* mutation and HPV. The considerable presence of *TERTp* mutation in HPV-positive tumors but a lack of statistical association between them have been previously described in the oral, oropharyngeal, and uterine cervical cancers, where all HPV16-positive oral SCCs and 70% of HPV-positive uterine cervical cancers harbored *TERTp* mutations [14, 22]. Some previous studies assumed that HPV-induced *TERTp* activations would be functionally different from the consequences of *TERTp* mutation in the absence of HPV [11, 18–20].

This study thoroughly investigated HPV status and genotypes in clinical specimens and stratified patients according to HPV status, with long-term follow-up data. In the present study, only high-risk HPV was dominated (80%) with the majority (92.2%) of HPV16. High-risk HPVs are the cause of approximately 31% of oropharyngeal SCCs [2, 9]. The prevalence of HPV infections in TSCCs is 37–80% in western countries and 35–73% in Korea [3, 7, 21, 32, 33].

HPV16 is the dominant virus in oropharyngeal SCC accounting for 82–87% of all HPV-positive cases [9]. The fraction of oropharyngeal cancer attributable to HPV is similarly the highest (> 40%) in developed countries including Europe, North America, Australia, New Zealand, Japan, and Republic of Korea [9, 34]. HPV infection was not an independent prognostic factor for OS and DFS in the 8th edition in the present study, which may be because of the incorporation of HPV into T and N categories of the staging system, and the effect of HPV might have been overshadowed by the impact of the staging modification because our study analyzed all together with HPV-positive and HPV-negative tumors.

In conclusion, the present study reveals that the *TERTp* mutation is present in a subpopulation of patients with TSCC and emphasizes the negative prognostic impact of *TERTp* mutation in tonsil cancers under the newly 8th edition of the AJCC staging system for oropharyngeal cancers, especially on HPV-negative TSCCs. This may be utilized to determine clinical aggressiveness of TSCCs.

## Data Availability

The data used to support the findings of this study are available from the corresponding author upon request.

## Abbreviations

TSCC: Tonsillar squamous cell carcinoma
HPV: Human papillomavirus
SCC: Oropharyngeal squamous cell carcinoma
AJCC: American Joint Committee on Cancer
*TERTp*: *TERT* promoter
OS: Overall survival
DFS: Disease-free survival
BOT: Base of tongue
